# A dye sensitized solar cell using natural counter electrode and natural dye derived from mangosteen peel waste

**DOI:** 10.1038/srep15230

**Published:** 2015-10-13

**Authors:** Wasan Maiaugree, Seksan Lowpa, Madsakorn Towannang, Phikun Rutphonsan, Apishok Tangtrakarn, Samuk Pimanpang, Prapen Maiaugree, Nattawat Ratchapolthavisin, Wichien Sang-aroon, Wirat Jarernboon, Vittaya Amornkitbamrung

**Affiliations:** 1 Department of Physics, Faculty of Science, Khon Kaen University, Khon Kaen 40002, Thailand; 2 Integrated Nanotechnology Research Center, Khon Kaen University, Khon Kaen 40002, Thailand; 3 Chumchon Ban Phon Ngam School, Akat Amnui District, Sakon Nakhon 47170, Thailand; 4 Nanotec-KKU Center of Excellence on Advanced Nanomaterials for Energy Production and Storage, Khon Kaen University, Khon Kaen 40002, Thailand; 5 Department of Chemistry, Faculty of Engineering, Rajamangala University of Technology Isan, Khon Kaen Campus, Khon Kaen 40000, Thailand

## Abstract

Mangosteen peel is an inedible portion of a fruit. We are interested in using these residues as components of a dye sensitized solar cell (DSSC). Carbonized mangosteen peel was used with mangosteen peel dye as a natural counter electrode and a natural photosensitizer, respectively. A distinctive mesoporous honeycomb-like carbon structure with a rough nanoscale surface was found in carbonized mangosteen peels. The efficiency of a dye sensitized solar cell using carbonized mangosteen peel was compared to that of DSSCs with Pt and PEDOT-PSS counter electrodes. The highest solar conversion efficiency (2.63%) was obtained when using carbonized mangosteen peel and an organic disulfide/thiolate (T_2_/T^−^) electrolyte.

Dye-sensitized solar cells (DSSCs) are a type of solar cell that has been intensively studied. Interest in DSSCs arises from their simple structure, low fabrication costs and promising efficiency in converting solar energy to electricity. This technology was first developed by O’Regan and Grätzel in 1991^1^. Three main components of a DSSC are the working electrode (WE), redox couple electrolyte (EL), and counter electrode (CE). Normally, the WE is composed of titanium dioxide attached with ruthenium complex dye (N719) coated upon a transparent conductive oxide. A triiodide/iodide (I_3_^-^/I^-^) redox couple EL is normally used in DSSCs. Recently, an organic disulfide/thiolate (T_2_/T^−^) solution was used as an electrolyte in a DSSC to take advantage of this electrolyte’s high transmittance and low corrosiveness^2^,^3^. Regarding the counter electrode material, Pt film is still widely used as a catalyst in DSSC devices. Unfortunately, use of Pt may not be a suitable option because of its high cost. To fabricate inexpensive solar cells, it is desirable to substitute low-cost catalytic materials for Pt. Such materials include carbon black^4^, carbon nanotubes^5^, graphite^6^, graphene^7^,^8^ and conductive polymers^9^,^10^,^11^,^12^. The catalytic activity of different carbon based electrodes was previously compared to Pt in T_2_/T^−^ organic electrolyte. It was found that the catalytic activity of T_2_/T^−^ match that of carbon based electrodes. Their surface areas are also high, therefore, the efficiency of carbon based DSSC was generally higher than Pt-DSSC’s^13^,^14^,^15^. In addition, replacement of ruthenium complex compounds by natural dyes derived from wood, flowers or fruits are alternative ways to reduce costs of these cells. Natural pigments extracted from roselle, blue pea flowers, shisonin, red Siahkooti fruit, red turnip and *Hibiscus surattensis* have been used as sensitizers in DSSCs. Unfortunately, all had solar to electric conversion efficiencies less than 2%^16^,^17^,^18^,^19^,^20^,^21^,^22^. However, there are reports of DSSCs sensitized using natural dyes attaining efficiencies exceeding 2%^23^,^24^,^25^. For example, Ito *et al.* used yellow pigment extracted from a *Monascus* (red rice) fermentation and reported an efficiency of 2.3%^26^.

Mangosteen (*Garcinia mangostana L.*) is a tropical fruit in Southeast Asia, and is known as “the queen of fruit” in Thailand. The mangosteen peel instantly becomes waste after being consumed. In the current study, we fabricated the three components of a DSSC from organic materials. Both dye and counter electrode were prepared from waste mangosteen peel (the structure of mangosteen peel DSSC shows in Fig. 1a). In this report, we focused upon the effects of the wrinkled honeycomb-like structure and nanoscale rough surface of mangosteen peel carbon counter electrodes upon dye-sensitized solar cell performance. The resulting DSSC was easy to fabricate and showed promisingly efficiency of 2.6% for natural dyes.

## Experimental

### Preparation of dye solution

Dye derived from mangosteen was obtained in the following manner. Fresh mangosteen peels were milled and dried for 3–5 days at room temperature. A ten gram sample of mangosteen peel powder was soaked in 100 mL acetone (1:10 ratio) with stirring for 12 h at room temperature. The crude solution was filtered using Whatman No. 41 filter paper to remove solid residue. Finally, the concentrated dye solution was shielded from exposure to direct light and stored in a refrigerator at 5 °C.

### Preparation of working electrode

TiO_2_ film was prepared as previous reported^27^. Fluorine doped tin oxide on glass (FTO glass, 8Ω/sq, Solaronix) was used as a substrate. The FTO glass was treated with an aqueous solution of TiCl_4_ (40 mM) at 70 °C for 30 min. Transparent and scattered TiO_2_ films, each with an area of 0.25 cm^2^, were coated on the TiCl_4_ layer by a screen printing technique using TiO_2_ pastes, PST-18NR and PST–400 C, respectively (Catalysts & Chemicals Ind. Co., Ltd). The details of TiO_2_ PST-18NR and TiO_2_ PST–400 C are shown in supporting information (Fig. S1). TiO_2_ films were annealed at 500 °C for 1 h then immersed in a concentrated dye solution for 24 h at room temperature.

### Preparation of counter electrode

Dried mangosteen peels were carbonized at 850 °C for 2 h in an argon atmosphere. Pieces of mangosteen peel carbon (MPC) were cut into slices with dimensions of 0.4 × 1.0 × 0.02 cm^3^. The slices of MPC were attached to FTO glass with10 μL of poly (3,4-ethylendioxythiophene)-poly (styrene sulfonate) (PEDOT-PSS). The MPCs attached to FTO glass were dried at ~80 °C for 6 h. A Pt counter electrode was prepared using a sputtering method and the PEDOT-PSS counter electrode was prepared using a drop casting method.

### Preparation of electrolyte solution

Liquid organic T_2_/T^−^ electrolyte was prepared from a mixture of 0.40 M C_4_H_6_N_8_S_2_ (T_2_ (di-5-(1-methyltetrazole)), prepared as detailed in ref. 2, 0.40 M C_6_H_15_N_5_S (NMe_4_^+^T^−^(5-mercapto-1-methyltetrazole N-tetramethylammonium salt)), prepared as detailed in ref. 2, 0.50 M TBP (4-tert-butylpyridine) and 0.05 M LiClO_4_ (lithium perchlorate) in acetonitrile solvent.

The liquid I_2_/NaI electrolyte was a mixture of 0.05 M iodide (I_2_), 0.5 M sodium iodide (NaI) and 0.0025 M lithium carbonate (Li_2_CO_3_) in acetonitrile.

### DSSC assembly

The DSSC was assembled using TiO_2_ coated mangosteen peel dye sensitizer film as the working electrode and MPC, PEDOT-PSS or Pt films as the counter electrode. These two electrodes were assembled using a semi-closed DSSC method. The electrolyte was filled into the cells.

### Film characterization

Film morphology was characterized using field emission scanning electron microscopy (FESEM; JEOL JSM-7001 F, Japan). Their structures were characterized using transmission electron microscopy (TEM) and selected area electron diffraction (SAED) (TECNAI G^2^, the Netherlands), respectively. The surface area of mangosteen peel carbon was determined using a Brunauer–Emmett–Teller method (BET, Quantachrome AS-1, USA). The chemical bonding of MPC was investigated using a micro Raman triple spectrometer, Jobin Yvon Model T64000, equipped with a green argon ion laser (514.32 nm, 30 mW). A laser beam with a diameter of about 2 microns illuminated MPC with total scan time about 5 mins. This triple spectrometer has a spectral resolution of ∼ 0.15 cm^−1^.

The electrical resistivities of films were determined using a four-point probe method (Keithley 617 Programmable Electrometer, USA) with Ag paste contacts. The film catalytic activity with T_2_/T^−^ was measured using a cyclic voltammeter (CV, Gamry REF 3000, U.S.A) in a three-compartment cell with a scan rate 20 mV/s in 10 mM NMe_4_^+^T^−^, 1 mM T_2_ and 0.1 M LiClO_4_ in an acetonitrile solution. A Pt plate and an Ag/AgCl electrode were used as a counter electrode and reference electrode, respectively. The cell performance was measured using a solar simulator (Peccell, PE-L111, Japan) system with a light intensity of 100 mW·cm^−2^. Incident photon-to-electron conversion efficiencies (IPCEs) of the devices under short-circuit conditions were determined with the aid of a Xe lamp (Oriel 150 W, USA) fitted with a monochromator (Cornerstone TM 130 1/8 m, USA) to provide a monochromatic light source. A silicon photodiode (Newport 818-UV, USA) was used for power density calibration with a picoammeter (Keithley 6485, USA). DSSC impedance was measured using electrochemical impedance spectroscopy (EIS, Gamry REF 3000, USA) under a light intensity 100 mW·cm^−2^ with frequency varied from 0.05 Hz to 100,000 Hz and an AC amplitude of 10 mV.

## Results and Discussion

**Figure 1:**
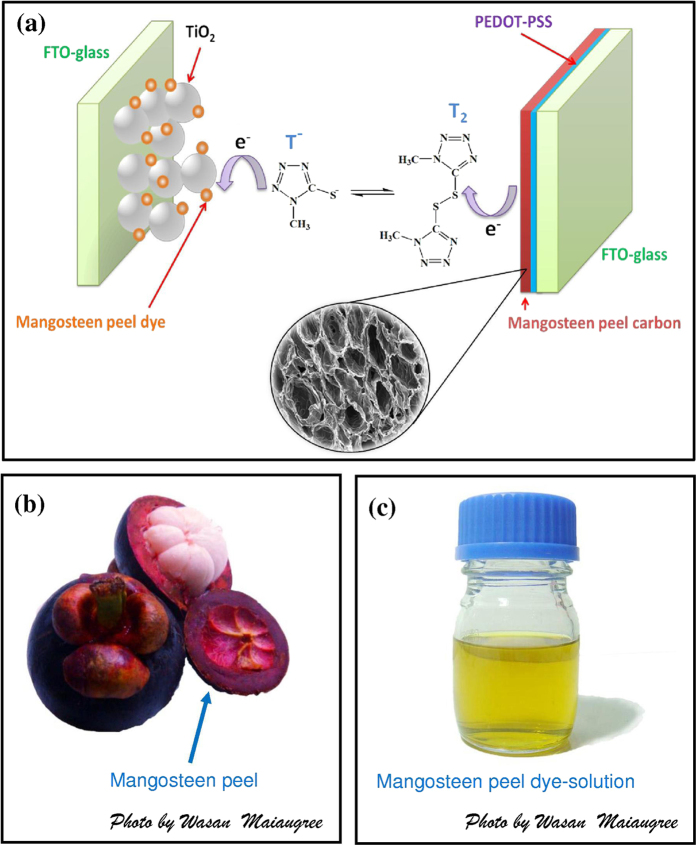
(**a**) The structure of mangosteen peel DSSC, (**b**) photograph of mangosteen (Photo by Wasan Maiaugree), and (**c**) mangosteen peel dye-solutions (Photo by Wasan Maiaugree).

**Figure 2:**
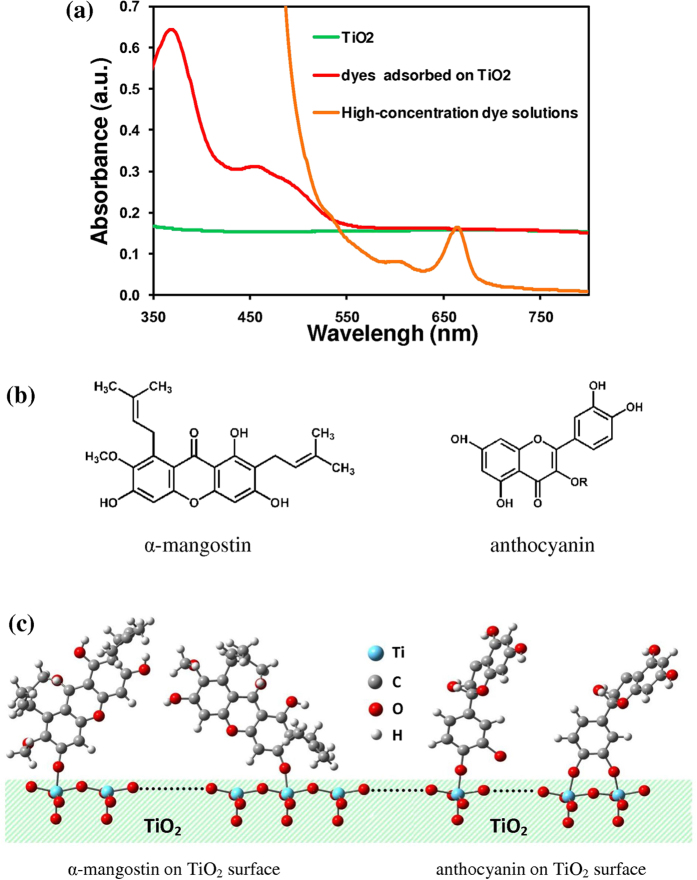
(**a**) The absorbance spectra of the mangosteen peel dye solutions, TiO_2_ and the desorbed dye solutions of the TiO_2_/Dye film in 5 ml of 0.1 M NaOH and ethanol at volume ratio of 1:1 and (**b**) structures of α-mangostin and anthocyanin and (**c**) possible binding schemes of α-mangostin and anthocyanin to TiO_2_.

Figure 1b,c shows an optical image of mangosteen and concentrated dye solution extracted from mangosteen peel. A transparent yellow dye solution was obtained after filtration. The main components in the mangosteen peel extract were α-mangostin and anthocyanin derivatives^22^,^28^,^29^,^30^. The presence of these derivatives was revealed through their light absorption characteristics derived from UV-vis spectra. The absorbance spectra of solutions containing highly concentrated dye extended all the way from 350–680 nm (Fig. 2a) where the signature peak of chlorophyll was also found at 610 and 665 nm^31^. However, the absorption spectrum of mangosteen peel dye on TiO_2_ showed absorption at wavelengths ranging from 350 to 550 nm. In this case, chlorophyll peak disappeared because there was little chlorophyll on TiO_2_. Furthermore, a peak for α-mangostin was observed at around 350–370 nm^30^,^32^ whilst anthocyanin derivative peaks were identified at ∼440–460 nm^22^ and 530–550 nm^31^. The α-mangostin and anthocyanin derivatives (Fig. 2b) extracted from various parts of different plants were previously tested as photosensitizers for DSSCs^22^,^33^,^34^. It is notable that the absorption spectrum of the dye-on-TiO_2_ specimen was situated on a non-zero absorbance which belongs to TiO_2_ nanoparticles. The non-zero absorbance of TiO_2_ which occurs in Fig. 2a was assumed as an effect from a diffused reflection of suspending TiO_2_ nanoparticles in solvent. Natural pigments can form bonds with the oxygen site of TiO_2_ with the aid of carbonyl (C = O) and hydroxyl (O-H) groups^35^,^36^,^37^. Figure 2c illustrates how α-mangostin or anthocyanin could possibly bind with TiO_2_. For α-mangostin, a possible anchoring mode is monodentate coordination via its available hydroxyl group whereas anthocyanin’s possible anchoring mode is either by monodentate or bidentate bridging via its hydroxyl and carbonyl groups. For any successful bridging, when an incident photon is absorbed by α-mangostin and anthocyanin, electrons are promoted from a HOMO to LUMO state. Subsequently, the electron will be injected into the conduction band of the TiO_2_ through functional anchoring groups.

**Figure 3:**
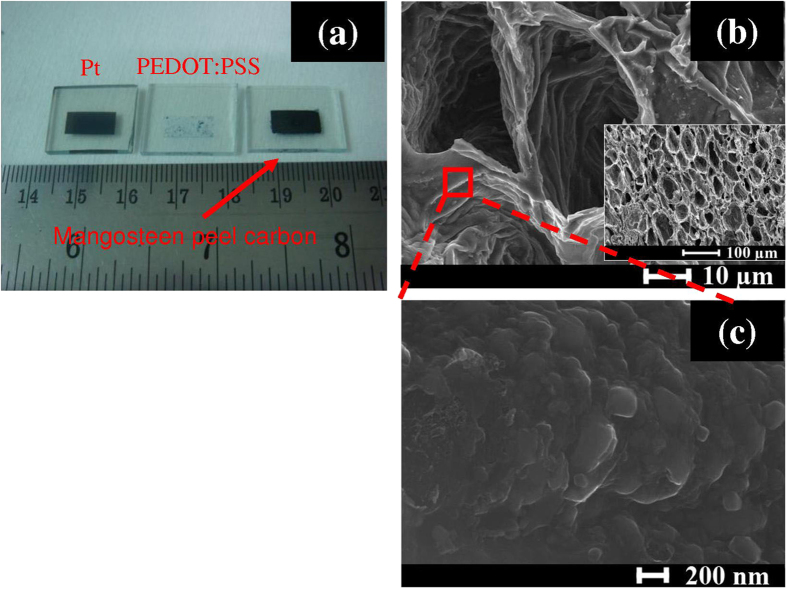
(**a**) Photograph of mangosteen peel carbon (MPC), PEDOT-PSS and Pt CEs, and (**b**,**c**) SEM images of mangosteen peel carbon (MPC).

Figure 3a shows images of Pt, PEDOT-PSS and MPC counter electrodes. The microstructure of MPC was characterized using FE-SEM and is shown in Fig. 3b,c. A honeycomb-like structure was observed with undulated walls as is seen in Fig. 3b. Moreover, the wall surface reveals some irregular nanoscale protrusions at high magnification as depicted in Fig. 3c. This created an unusually high interfacial area between the electrolyte and the counter electrode, which is excellent for DSSC applications. The thicknesses of MPC, Pt and PEDOT:PSS counter electrodes were 200 μm, 100 nm and 200 nm, respectively.

**Figure 4:**
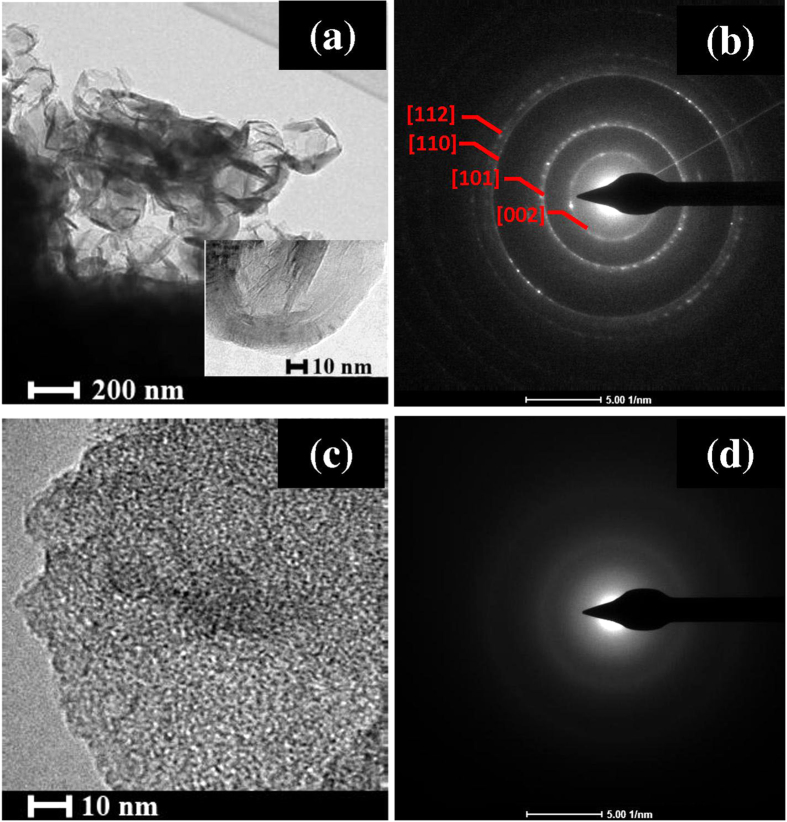
TEM images (**a**–**d**) and SAED patterns (e and f) of mangosteen peel carbon (MPC).

**Figure 5:**
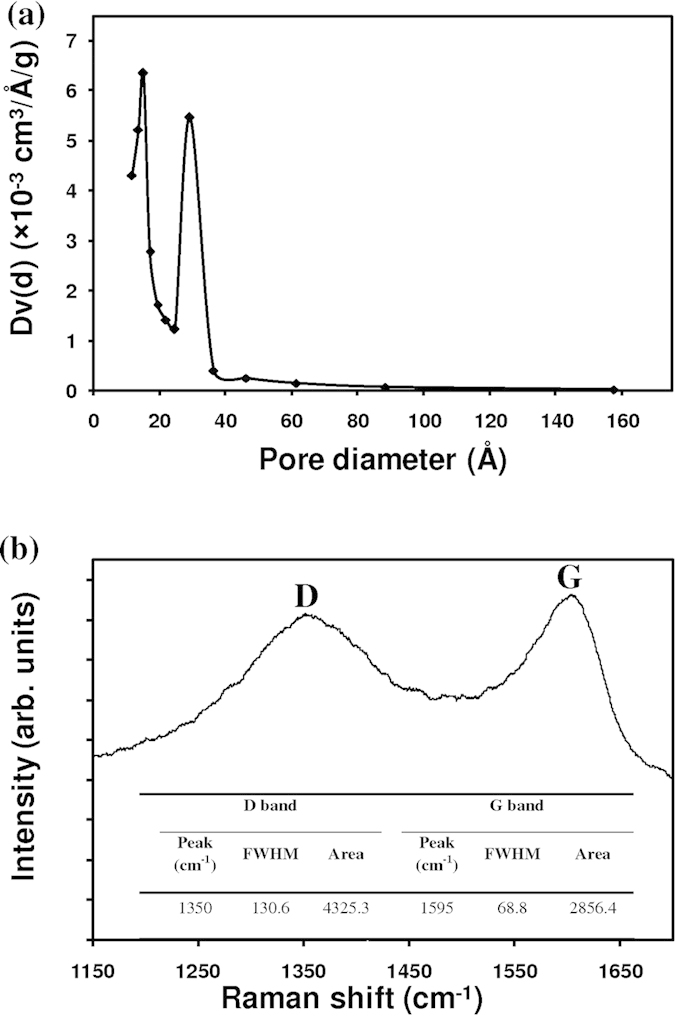
(**a**) Pore distribution and (**b**) Raman spectra of mangosteen peel carbon.

TEM images of MPC are presented in Fig. 4. MPC consists of graphite oxide nanosheets (Fig. 4a) and an amorphous carbon phase (Fig. 4c). The selected area electron diffractions of mangosteen peel carbon (Fig. 4b) are consistent with a graphite 2 H structure (JCPDF #751621) with the presence of (101), (110) and (112) planes. The enlargement of (002) d-spacing to 3.48 Å compared to that of graphite 2 H (3.40 Å), indicating the presence of graphite oxide. The amorphous part shows a diffuse diffraction pattern (Fig. 4d). The surface area of MPC was determined using a BET technique. MPC had 125 m^2^/g of surface area, which is higher than that of a mesoscopic carbon electrode 91.08 m^2^.g^−1^ 34, glassy carbon (10 m^2^.g^−1^)^6^, and graphite (10 m^2^.g^−1^)^6^, but is a slightly lower than that of carbon black electrode (163.9 m^2^.g^−1^)^38^, and much lower than graphite/carbon black (247.68 m^2^.g^−1^)^39^ and activated carbon (2000 m^2^.g^−1^)^6^. Though increasing the surface area of the mangosteen peel can be done by chemical activation and could contribute to a further increase of the DSSC efficiency. Such attempt was not made here in order to avoid the use of harmful chemicals and to keep the fabrication steps as simple as possible. The pore distribution within MPC was determined by a Barrett-Joyner-Halenda method (BJH) and is shown in Fig. 5a. The effective pore size distributions of MPC were in the range of 1.15 nm to 15.76 nm, and the average pore size was about 1.49 nm. However, the surface area of Pt and PEDOT-PSS films could not be measured due to an insufficient supply of materials for the BET measurement.

Figure 5b shows Raman spectra of MPC. A broad D-peak (130.6 cm^−1^ of FWHM) was located at 1350 cm^−1^ which indicated the high disorder of sp3 carbon. The narrower G peak (68.8 cm^−1^ of FWHM) at 1595 cm^−1^ correlated with a graphite oxide phase, as indicated in previous published work^40^. From this information, it is clear that graphite oxide from MPC is a highly ordered sp2 hexagonal carbon oxide network.

The electrical resistivity (ρ) of PEDOT-PSS, MPC and Pt films on their glass substrates were measured using a four-point probe method. The results are given in Table 1. The electrical resistivity of PEDOT-PSS was 1.57 × 10^−2^ Ω•m and its electrical conductivity (σ) was 6.38 × 10 S/m. In the case of MPC and Pt films, low electrical resistivities of 1.72 × 10^−4^ Ω•m and 1.46 × 10^−5^ Ω•m were observed, respectively. Consequently, high σ values were observed, 5.81 × 10^3^ S/m for MPC and of 6.85 × 10^4^ S/m for Pt. This suggests that an electron can easily move from FTO glass to the front surface of the film (in contact with electrolyte).

**Figure 6:**
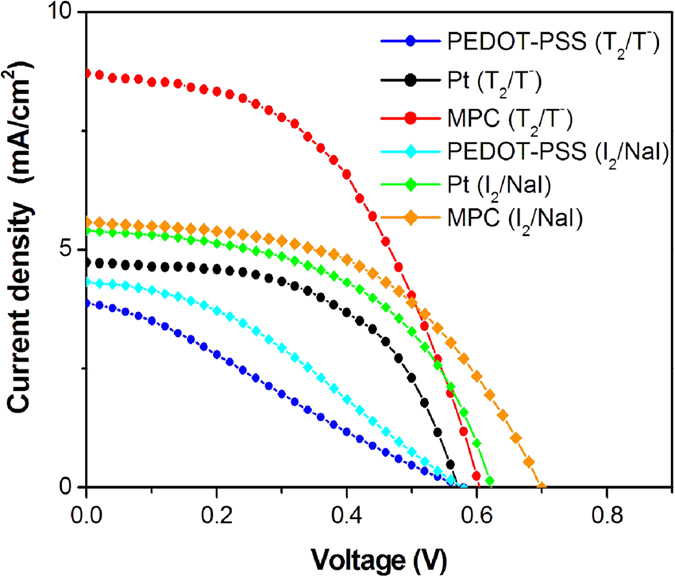
Photocurrent density (J) vs. photovoltage (V) curves of mangosteen peel carbon (MPC), PEDOT-PSS and Pt DSSCs based on T_2_/T^−^ and I_2_/NaI electrolytes.

The photocurrent density-voltage characteristics of MPC, PEDOT-PSS and Pt DSSCs based on T_2_/T^−^ and I_2_/NaI electrolytes are illustrated in Fig. 6. The photovoltaic performances of DSSCs are listed in Table 2. In the case of T_2_/T^−^, the MPC based DSSC had an open-circuit voltage (V_OC_) of 0.60 V, a short-circuit current density (J_SC_) of 8.70 mA∙cm^−2^, a fill factor (FF) of 0.50, and conversion efficiency (η) of 2.63%. In the case of the Pt based DSSC, values of V_OC_, J_SC_, FF and η were 0.57 V, 4.72 mA∙cm^−2^, 0.54 and 1.47%, respectively. Values of 0.58 V, 3.88 mA∙cm^−2^, 0.27 and 0.6% were observed for V_OC_, J_SC_, FF and η, respectively, for the PEDOT-PSS based DSSC. The short-circuit current density and conversion efficiency of MPC based DSSC were higher than those of the typical Pt and PEDOT-PSS based DSSCs. In the case of I_2_/NaI electrolyte, the magnitude of performance values follows the order: MPC (1.99%) >Pt (1.75%) >PEDOT-PSS (0.88%). This trend is similar to T_2_/T^−^ base electrolyte, but the highest efficiency (MPC-I_2_/NaI) was lower than the efficiency of MPC-T_2_/T^−^. Therefore, we were interested only in organic T_2_/T^−^ electrolyte. To confirm the effect of counter electrode type upon the DSSC performance, commercial N719 dye was used as a sensitizer in the working electrode and disulfide/thiolate was the redox couple electrolyte. DSSCs using N719 dye showed the same trend as the mangosteen peel dye DSSC. The details are shown in supporting information (Fig. S2 and Table S1).

**Figure 7:**
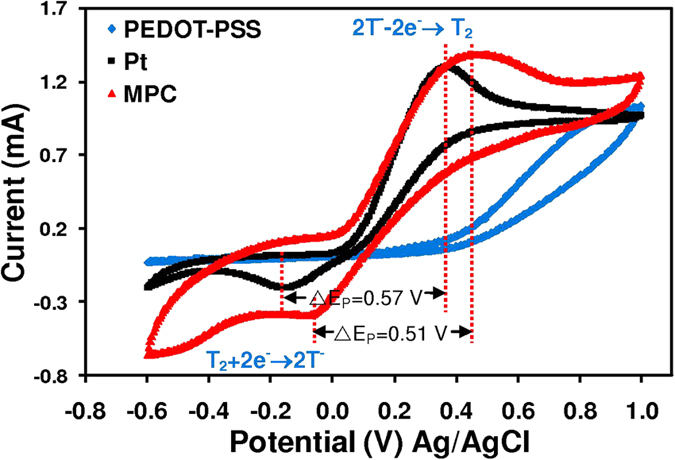
Cyclic voltammogram (CV) curves of mangosteen peel carbon (MPC), PEDOT-PSS and Pt electrodes at a scan rate of 20 mV/s in 10 mM NMe_4_^ + ^T^−^, 1 mM T_2_ and 0.1 M LiClO_4_ in an acetonitrile solution.

To investigate the catalytic activity of the counter electrodes in the reduction of T_2_, redox properties of the three electrodes were determined using cyclic voltammetry (CV). Figure 7 shows the CV curves of PEDOT-PSS, MPC electrodes and Pt electrodes. Two typical redox peaks were clearly observed for MPC and Pt electrodes, indicating high catalytic activity of T_2_ reduction. A relative negative pair was associated with the reduction reaction of T_2_ + 2e^*−*^ *→* 2T^−3^, whereas a positive peak was attributed to the oxidation reaction of 2T^−^ − 2e^−^ *→* T_2_. It was found that the MPC counter electrode exhibited a higher absolute current peak and more positive reduction peak potential than did the Pt and PEDOT-PSS counter electrodes. Moreover, the difference between the potential value of the oxidation peak and the reduction peak (ΔE_P_) of Pt is 57 mV, whilst MPS’s was 51 mV. Since ΔE_P_ was inversely correlated with the electron transfer rate constant (k_S_), that is, a smaller ΔE_P_ indicates higher k_S_^41^,^42^. Therefore, the MPC film manifested a faster reduction reaction rate in T_2_ electrolyte. In comparison with another carbon-based counter electrode under the same conditions, the ΔE_P_ in current study was smaller than in another study based on annealed hydrothermal carbonized-glucose/TiC composite (59 mV)^43^. The excellent CV of MPC is likely due to its large surface area, PEDOT-PSS co-catalyst (binder) and mangosteen peel carbon. However, a poor redox reaction rate was observed for pure the PEDOT-PSS electrode in comparison to the MPC electrode. This indicated that the electrocatalytic activity of the MPC electrode can be attributed to MPC. Larger electrochemical-catalytic activity of MPC is advantageous for its efficient performance in the counter electrode of this DSSC device.

**Figure 8:**
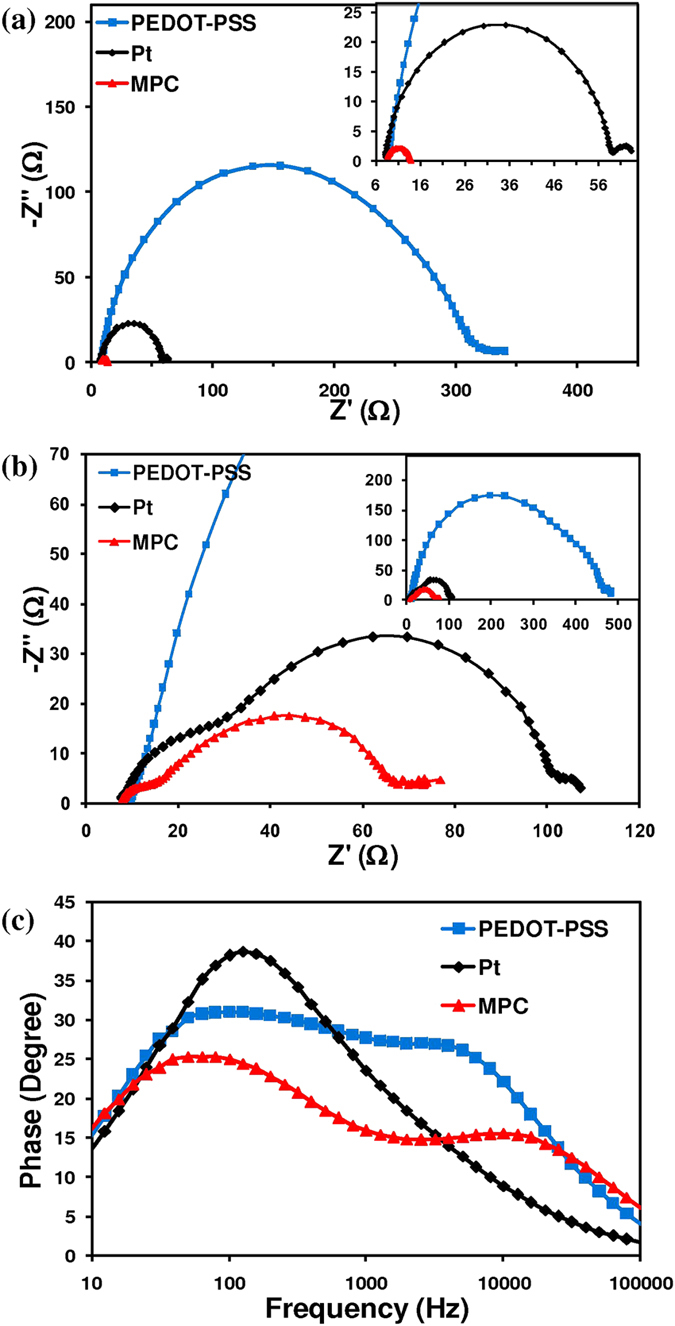
Nyquist plots of mangosteen peel carbon (MPC), and Pt electrodes for (a) symmetrical cell (CE-CE) (b) dye sensitized solar cells (DSSC) and (c) bode plot of DSSCs with MPC, PEDOT-PSS and Pt CE based on organic T_2_^/^T^−^ electrolytes.

**Figure 9:**
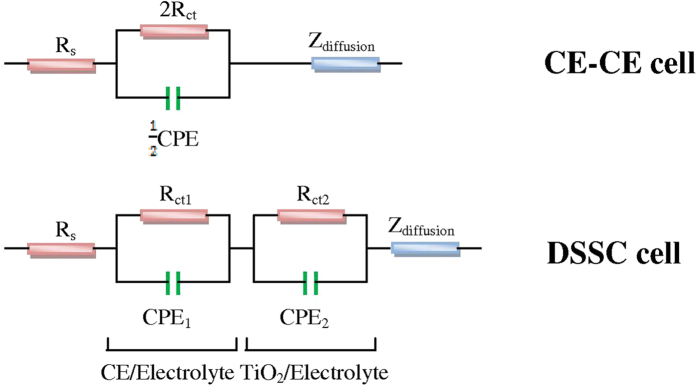
Schematic of (CE-CE) and DSSC equivalent circuit based on organic T_2_/T^−^ electrolytes.

To reveal the electrochemical characteristics of DSSC counter electrodes, electrochemical impedance spectra (EIS) were measured in a symmetric cell configuration. This consisted of two identical counter electrodes (CEs). As shown in Fig. 8a, Nyquist plots of the CE–CE cells exhibit two semicircles. The semicircle in the high frequency region (left semicircle) is related to the charge-transfer resistance (R_ct_) of the counter electrode/redox (T_2_/T^−^) electrolyte interface. The semicircle in the low frequency region (right semicircle) represents the Nernst diffusion impedance (Z_N_) of the T_2_/T^−^ redox couple within the electrolyte. The equivalent circuit of CE–CE cells obtained from fitting EIS spectra is shown in Fig. 9. R_ct_ is of primary interest as it represents the electron flow during the reduction of T_2_ at the counter electrode. It is known that small resistance allows faster electron transfer rate, resulting in a higher solar cell performance. Larger resistance suppresses electron flow and causes low solar cell efficiency. In the case of the MPC symmetrical counter electrode, a small semicircle was observed in the high frequency region. The charge-transfer resistance of the MPC electrode (3.42 Ω) was lower than that of typical Pt (25.25 Ω) and PEDOT-PSS electrodes (152.25 Ω). This suggests that the MPC electrode has a superior electrocatalytic activity for the redox reaction of the T_2_/T^−^ couple and a faster electron transport. These properties are important for improving the photovoltaic performance of the MPC based DSSC. The simulated chemical capacitance (C_μ_) of the MPC electrode (47.16 μF) was significantly larger than that of the Pt electrode (11.28 μF). Provided that a counter electrode exhibits a capacitive property of an electrical double-layer capacitor^44^, the capacitance value should related to the surface area of counter electrode. That is, a large electrochemical capacitance value of the electrode should relate to a large specific surface area, which is a crucial factor for high electrocatalytic activities^45^,^46^,^47^. In this study, an MPC electrode had a higher active surface area than that of sputtered Pt because its capacitance value was larger than Pt’s. This is because the honeycomb-like features with nanoscale rough surfaces provide a large surface area in MPC electrodes. EIS of DSSCs was determined and is shown in Fig. 8b. Three semicircles were found in the EIS data for these DSSC devices. The high frequency region (left semicircle) represents the charge-transfer resistance at the counter electrode/electrolyte interface (R_ct1_). The middle frequency region (middle semicircle) relates to the charge-transfer resistance at the working electrode/electrolyte interface (R_ct2_). The low frequency region (right semicircle) represents the electrolyte diffusion resistance (Z_N1_). The series resistance R_s1_, R_ct1_ and R_ct2_ values of three counter electrodes are fitted and summarized in Table 1. It is seen that R_ct1_ of MPC based DSSC was 5.41 Ω, which is smaller than those of Pt based DSSC (19.50 Ω) and PEDOT-PSS based DSSC (332.34 Ω). This result agrees well with the EIS outcomes of symmetrical CE-CE sets.

**Figure 10:**
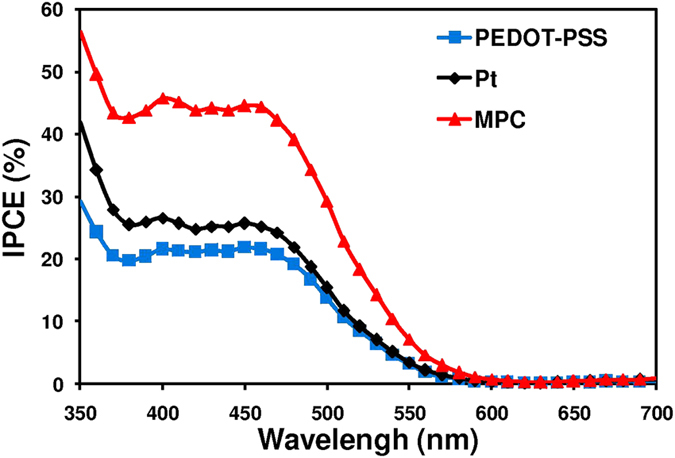
Incident photon-to-collected electron conversion efficiencies (IPCE) spectra of mangosteen peel carbon (MPC), PEDOT-PSS and Pt DSSCs based on organic T_2_^/^T^−^ electrolytes.

To confirm the validity of the J_SC_ trend derived from the I-V curve, the incident photon-to-collected electron conversion efficiencies (*IPCE*) were measured since J_SC_ is related to IPCE values according to Eq. 1:^48^,^49^


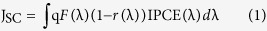


where 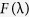 is the incident photon flux density, 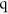 is the electronic charge and 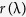 is the incident light loss in light absorption and reflection by the FTO-glass. The large 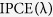 was related to high 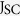 in the DSSC. The IPCE spectra of the DSSCs based upon organic T_2_/T^−^ electrolytes are shown in Fig. 10. The spectra present an effective absorption of mangosteen peel dye in comparison with other counter electrodes. All IPCE spectra were measured in wavelengths of 380–560 nm. The DSSC based upon a MPC counter electrode showed higher IPCE values than those of Pt and PEDOT-PSS based DSSCs. The maximum possible of J_sc_ values calculated from IPCE were 9.66 mA.cm^−2^ for MPC DSSC, 5.87 mA.cm^−2^ for Pt DSSC and 4.67 mA.cm^−2^ for PEDOT DSSC. This trend was similar to that of J_sc_ values obtained from the I-V curve.

The improvement of J_SC_ results from superior electron transportation at the counter electrode/electrolyte interfaces and good electrocalytic activity of MPC electrode. It is the key that leads to the high efficiency of the MPC based DSSC. The nanoscale roughness and honeycomb-like morphology of MPC electrode provide large surface area and high electrocatalytic activity for T_2_/T^−^ redox reaction as evidenced in Figs 7 and 8a,b.

## Conclusions

This work reports the preparation and photovoltaic performance of a natural organic- based DSSC. Dye extracted from mangosteen peel and carbonized mangosteen peel film were employed as a photosensitizer and a counter electrode catalyst, respectively. An organic disulfide/thiolate mixture was used as the electrolyte. SEM and TEM images show that mangosteen peel carbon counter electrode had a large active surface area due to its wrinkled honeycomb like structure with a nano-rough surface. The efficiency of the DSSC sensitized by mangosteen peel dye extract and mangosteen peel carbon counter electrode was 2.63%. This was higher than that of Pt counter electrode (1.47%). These results clearly show facile applications of a natural dye and carbon electrode derived from mangosteen peel as a photosensitizer and counter electrode in a DSSC. It shows promise for the realization of high performance from natural, low cost and environmentally friendly photovoltaic cells made from organic waste.

## Additional Information

**How to cite this article**: Maiaugree, W. *et al.* A dye sensitized solar cell using natural counter electrode and natural dye derived from mangosteen peel waste. *Sci. Rep.* **5**, 15230; doi: 10.1038/srep15230 (2015).

## Supplementary Material

Supplementary Information

## Figures and Tables

**Table 1:** 

Counter electrode	Four-point probe		EIS of symmetric cells			EIS of DSSCs			
	ρ (Ω•m)	σ (S/m)	R_s_ (Ω)	R_ct_ (Ω)	C_μ_ (μF)	R_s1_ (Ω)	R_ct1_ (Ω)	ƒ_max_ (Hz)	τ (ms)
PEDOT-PSS	1.57 × 10^−2^	6.38 × 10	8.94	152.25	10.71	9.13	332.34	100	1.59
Pt	1.46 × 10^−5^	6.85 × 10^4^	8.15	25.25	11.28	8.24	19.50	126	1.26
MPC	1.72 × 10^−4^	5.81 × 10^3^	8.27	3.42	47.16	8.31	5.41	79	2.01

Summary of electrical resistivity (ρ) and conductivity (σ) from a four-point probe, series resistance (R_s_), charge-transfer resistance (R_ct_), capacitance (C_μ_) from symmetric cells, series resistance (R_s1_) and charge-transfer resistance (R_ct1_) at counter electrode/electrolyte, maximum frequency of the mid-frequency peak (ƒ_max_), electron lifetime (τ) of the DSSCs for mangosteen peel carbon (MPC), PEDOT-PSS and Pt DSSCs based on organic T_2_/T^−^ electrolytes.

**Table 2:** 

Counter Electrode	Electrolyte	J_SC_(mA cm^−2^)	V_OC_ (V)	FF	η (%)
Pt	T_2_/T^−^	4.72	0.57	0.54	1.47
PEDOT-PSS	T_2_/T^−^	3.88	0.58	0.27	0.60
Mangosteen peel carbon (MPC)	T_2_/T^−^	8.70	0.60	0.50	2.63
Pt	I_2_/NaI	5.40	0.62	0.52	1.75
PEDOT-PSS	I_2_/NaI	4.33	0.58	0.35	0.88
Mangosteen peel carbon (MPC)	I_2_/NaI	5.58	0.70	0.51	1.99

Photovoltaic characteristics of mangosteen peel dye DSSCs using different counter electrodes; open-circuit voltage (V_oc_), short-circuit current density (J_sc_), fill factor (FF) and solar cell efficiency (η).
