# Supplementary material for: A dye sensitized solar cell using natural counter electrode and natural dye derived from mangosteen peel waste
Source: Sci Rep. 2015 Oct 13;5:15230. doi: 10.1038/srep15230 (PMC4602286; doi:10.1038/srep15230)
Supplement: Supplementary Information [file srep15230-s1.pdf]

Supporting information

A dye sensitized solar cell using natural counter electrode and natural dye derived from mangosteen peel waste

Wasan Maiaugree,[†] Seksan Lowpa,[†] Madsakorn Towannang,[†] Phikun Rutphonsan,[†] Apishok Tangtrakarn,[†,‡,⊥] Samuk Pimanpang,[†,‡] Prapen Maiaugree,[§] Nattawat Ratchapolthavisin,[‡] Wichien Sang-aroon,[ǁ] Wirat Jarernboon[†] and Vittaya Amornkitbamrung*[,†,‡,⊥]

[†]Department of Physics, Faculty of Science, Khon Kaen University, Khon Kaen 40002, Thailand

[‡]Integrated Nanotechnology Research Center, Khon Kaen University, Khon Kaen 40002, Thailand

[§]Chumchon Ban Phon Ngam School, Akat Amnui District, Sakon Nakhon 47170, Thailand

[⊥]Nanotec-KKU Center of Excellence on Advanced Nanomaterials for Energy Production and Storage, Khon Kaen University, Khon Kaen 40002, Thailand

[ǁ]Department of Chemistry, Faculty of Engineering, Rajamangala University of Technology Isan, Khon Kaen Campus, Khon Kaen 40000, Thailand

*Corresponding Author's email: Vittaya@kku.ac.th

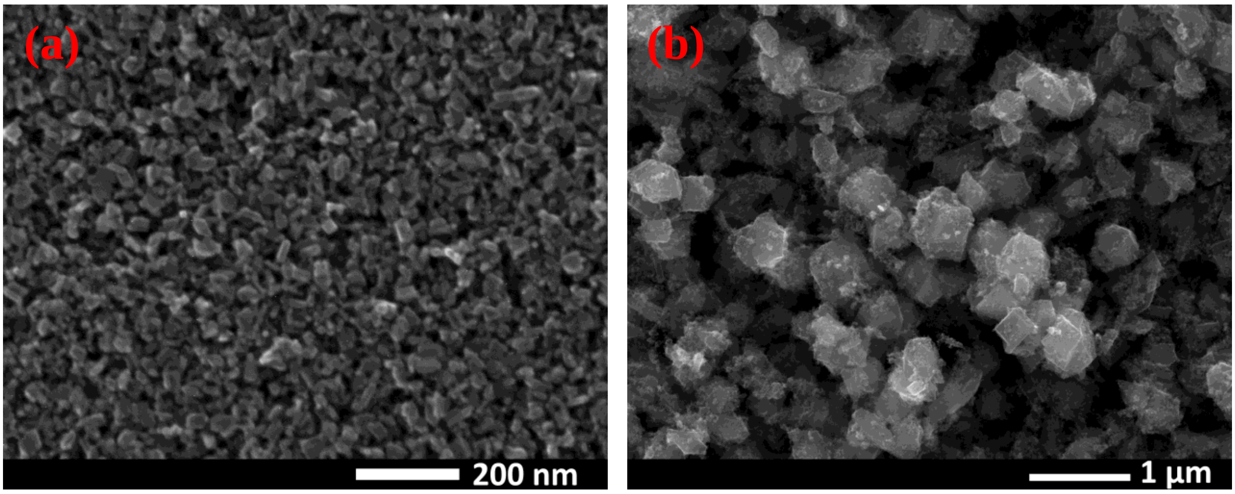

Fig. S1: FESEM images of (a) TiO_2 PST-18NR and (b) TiO_2 PST-400C.

The morphology of the transparent layer TiO_2 PST-18NR films in Fig. S1(a) indicates a uniform particle distribution on the FTO surface with nanoparticle diameters ~15-20 nm. Fig.S1(b) shows a scattering layer of TiO_2 PST-400C with a TiO_2 particle size of ~400 nm.

The photocurrent density-voltage characteristics of N719 dye DSSCs based upon MPC, PEDOT-PSS and Pt CEs with thiolate/disulfide electrolyte are illustrated in Fig. S2. The photovoltaic performance of N719 dyes DSSCs is listed in Table S1. The DSSC with a MPC counter electrode had an open-circuit voltage (V_{OC}) of 0.65 V, a short-circuit current density (J_{SC}) of 14.20 mA·cm^{-2}, a fill factor (FF) of 0.50, and conversion efficiency (η) of 4.65%. Compared to a typical Pt DSSC, it had the following photovoltaic parameters: V_{OC} = 0.64 V, J_{SC} = 9.26 mA·cm^{-2}, FF = 0.60 and η = 3.61%. PEDOT-PSS DSSCs, showed V_{OC} of 0.70 V, J_{SC} of 6.12 mA·cm^{-2}, FF of 0.44 and η of 1.92 %. It can be seen that the short-circuit current density and conversion efficiency of the MPC (T_2/T^-) DSSC was higher than those of the Pt and PEDOT-PSS DSSC. These results show the same trend as the mangosteen peel dye DSSC. However in this work, we would like to investigate a natural dye and counter from a waste material.

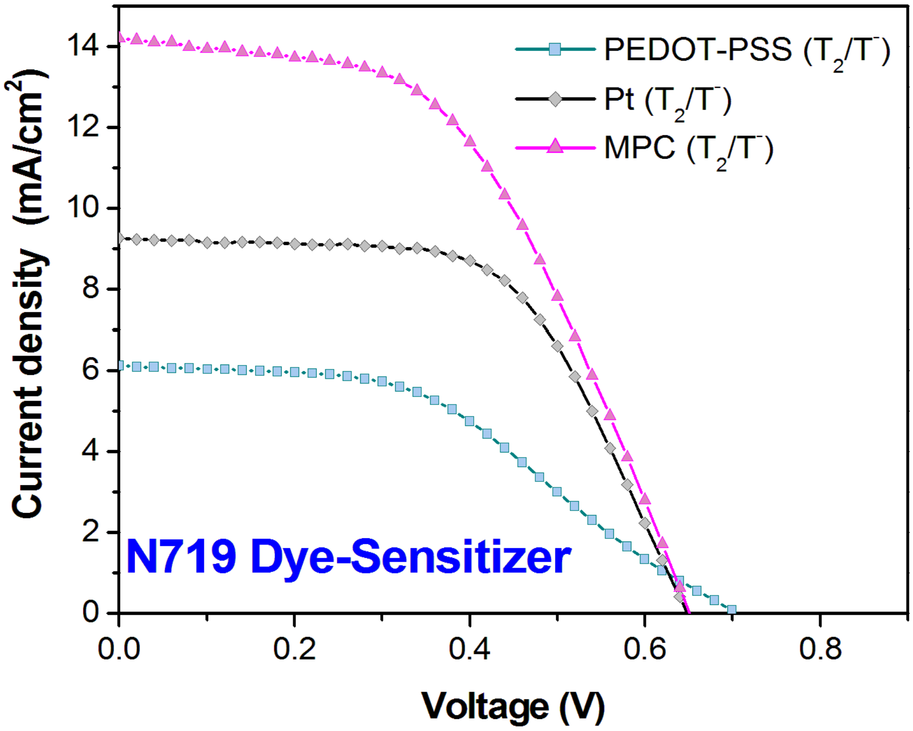

Fig. S2: Photocurrent density (J) vs. photovoltage (V) curves of N719-dye DSSCs using mangosteen peel carbon (MPC), PEDOT-PSS and Pt Counter electrodes based upon T_2/T^- electrolytes.

Table S1. Photovoltaic characteristics of N719 dye DSSCs using different counter electrodes based upon organic T_2/T^- electrolytes; open-circuit voltage (V_{oc}), short-circuit current density (J_{sc}), fill factor (FF) and solar cell efficiency (η).

**Counter Electrode**	**Dye-sensitizer**	**Electrolyte**	**J_{SC} (mA cm^{-2})**	**V_{OC} (V)**	**FF**	**η (%)**
Pt	N719	T_2/T^-	9.26	0.64	0.60	3.61
PEDOT-PSS	N719	T_2/T^-	6.12	0.70	0.44	1.92
Mangosteen peel carbon (MPC)	N719	T_2/T^-	14.20	0.65	0.50	4.65
